# Q-TriLSTM-Vision: a quantum-interference- augmented tri-stream LSTM for multi-label plant stress recognition on the OLID-I benchmark

**DOI:** 10.3389/fpls.2026.1903924

**Published:** 2026-07-31

**Authors:** Karthikeyan A, Sundar S

**Affiliations:** School of Electronics Engineering, Vellore Institute of Technology, Vellore, Tamil Nadu, India

**Keywords:** hybrid deep learning, multi-label classification, plant stress recognition, precision agriculture, quantum-inspired learning, tri-stream LSTM

## Abstract

Plant stress recognition plays a vital role in precision agriculture by enabling the early detection of diseases, insect infestations, and nutrient deficiencies that adversely affect crop productivity. Although deep learning models have achieved promising results, existing CNN-, Transformer-, and hybrid architectures often struggle to capture complex spatial dependencies, distinguish visually similar stress symptoms, and handle class imbalance in multi-label classification. To address these challenges, this paper proposes Q-TriLSTM-Vision, a novel hybrid deep learning framework that integrates an EfficientNet-B0 visual encoder, a tri-stream long short-term memory (TriLSTM) network, and a lightweight quantum-inspired interference gate. Unlike quantum computing-based approaches, the proposed interference mechanism is implemented entirely using classical neural operations to enhance feature representation without requiring quantum hardware. The model further employs an entanglement-inspired attention fusion module, focal binary cross-entropy loss with label smoothing, weighted sampling, and per-class threshold calibration to improve discriminative learning and minority-class recognition. The proposed framework was evaluated on the OLID-I dataset and further validated on the PlantVillage and PlantDoc benchmark datasets. Experimental results demonstrate that Q-TriLSTM-Vision achieved Macro-F1 scores of 0.9127, 0.9624, and 0.8975 on OLID-I, PlantVillage, and PlantDoc, respectively, outperforming representative CNN-, Transformer-, and hybrid deep learning models while achieving lower Hamming loss and improved recall. Cross-validation, ablation studies, and statistical significance analysis further confirm the robustness and effectiveness of the proposed framework. Overall, Q-TriLSTM-Vision provides an accurate, computationally efficient, and reliable solution for intelligent plant stress recognition in precision agriculture.

## 1 Introduction

Plants become stressed when they are exposed to conditions that hinder their growth and metabolic processes (Lichtenthaler, 1996). In cases where plants are overstressed by adverse environmental conditions, irreparable damage or even death may occur (Pahlich, 1993). Two broad categories of environmental conditions cause plant stress: biotic factors, which include living organisms such as fungi, bacteria, and insects, and abiotic factors, which include drought, salt stress, and nutrient deficiencies (Mosa et al., 2017). Plant stress factors have a major impact on the agricultural sector. The extent of losses suffered by crops due to stress factors ranges from 26.3% to 40.3% (Oreke, 2006). According to the research done, it is estimated that about 37 per cent of the agricultural yields are lost because of pests and pathogens, and 13 per cent of the yield is lost due to insects. Moreover, crop nutrition deficiencies pose a threat to more than two billion people’s food security (Gaikwad et al., 2020), causing a loss of yield up to 70 per cent (Francini and Sebastiani, 2019). The idea behind precision agriculture is that it allows for the utilization of superior resources and ensures continuous improvement in the food chain’s sustainability. The role played by precision agriculture in pest management and nutrient monitoring has been greatly illustrated through various studies (Nugroho et al., 2020; Lo Presti et al., 2023).

AI is continually increasing its significance due to its ability to introduce innovations and use strong tools to address difficult issues that regular computer software and humans find difficult to tackle. The growing adoption of AI does not constitute an exception to precision agriculture. Indeed, applications based on data play an essential role in this sphere. Machine vision technology, for instance, has a broad application in herbicide, livestock, and crop management (Dhanya et al., 2022). In order to learn and improve accuracy, AI relies heavily on abundant accessible data. However, in the case of precision agriculture, efforts and resources needed for data gathering, annotation, and laboratory testing prove to be tedious (Lu and Young, 2020). Open data, on the other hand, helps eliminate such difficulties, thus fostering research and ensuring replicability of results.

Initial experiments conducted by Mohanty et al. (2016) proved the capability of training a CNN model on the PlantVillage dataset for detection of plant diseases with more than 99% accuracy. Yet, the PlantVillage dataset was obtained with artificial uniform lighting in a laboratory environment, and it contains classes consisting of single label diseases only without reflecting multiple stressors common in agricultural fields.

As multi-label tasks differ significantly from their corresponding single-label counterparts, they present new problems not faced in the former. The presence of label dependencies, the occurrence imbalance of some joint labels, and the need for individual class decision thresholds render CNN pipelines ineffective (Sunil et al., 2023; Zhang et al., 2023). While Transformer-based vision models have shown some promise at handling long-range spatial dependencies (Dosovitskiy et al., 2021), their self-attention mechanisms with quadratic complexity lead to considerable computation costs for edge devices.

Recurrent models, particularly LSTMs, provide an effective sequential alternative to standard methods when images are processed with patch tokens (Graves and Schmidhuber, 2005). A bidirectional LSTM is capable of processing information in both left-to-right and right-to-left orders in the token sequence, and a third stream processing tokens in a zigzag order provides additional inter-token context not encoded in either stream. Drawing inspiration from quantum information theory, we develop a novel Quantum Interference Gate (QIG) for LSTM gating based on the superposition of projections to modulate gate behavior inspired by wave interference (Yang et al., 2023; Senokosov et al., 2024). This novel gate design adds no additional parameters to the network architecture while greatly enhancing sensitivity to early stress indicators.

The principal contributions of this paper are:

The principal contributions of this paper are as follows:

- A novel Q-TriLSTM-Vision architecture is proposed by integrating an EfficientNet-B0 feature extractor, a tri-stream LSTM, and a quantum-inspired interference gate for multi-label plant stress recognition.
- A lightweight quantum-inspired interference mechanism is introduced to enhance LSTM memory gating through classical interference-based feature modulation without requiring quantum hardware or quantum circuits.
- A tri-stream sequential learning strategy is developed to simultaneously capture forward, backward, and diagonal spatial dependencies among image patches, improving discrimination of visually similar plant stress symptoms.
- An entanglement-inspired attention fusion module is designed to effectively combine the three directional feature representations, resulting in enhanced feature learning and classification performance.
- A comprehensive optimization framework, incorporating focal binary cross-entropy loss, label smoothing, weighted random sampling, data augmentation, and per-class threshold calibration, is employed to improve robustness and address class imbalance.
- Extensive experimental validation on the OLID-I, PlantVillage, and PlantDoc datasets demonstrates that the proposed framework consistently outperforms representative CNN-, Transformer-, and hybrid deep learning models, with its effectiveness further confirmed through ablation studies, cross-validation, and statistical significance analysis.

The remainder of this paper is structured as follows: Section 2 reviews related work across plant stress detection, recurrent vision architectures, quantum-inspired neural mechanisms, and multi-label classification. Section 3 describes the OLID-I dataset and preprocessing pipeline. Section 4 presents the proposed Q-TriLSTM-Vision methodology in full technical detail. Section 5 reports experimental results, ablation studies, per-crop analysis, training dynamics, and computational benchmarks. Section 6 discusses implications, limitations, and future directions. Section 7 concludes.

## 2 Related work

Sunil and Jaidhar (2024) systematic review identified more than 200 deep learning models for plant disease detection, which showed that EfficientNet-B4 and ResNet-50 were the most consistent high-performing models in single label tasks.

Plant disease recognition in a multi-class setting that also incorporates biotic stress like pest infestation has been investigated by Zhou et al. (2024), and it was shown that attention-based techniques are very effective in discriminating between classes where there is high visual similarity between pests’ and diseases’ lesions. Detection of multiple types of stresses from a single leaf image, called the multi-label problem, has been first formalized by Orka et al. (2023) using their OLID-I data set, highlighting the shortcomings of existing approaches that analyze one type of stress at a time.

For the detection of nutritional deficiency, Bera et al. (2024) introduced PND-Net, a graph convolutional network, which uses plant leaf graph representations for modeling nutrient-deficiency symptoms and obtained competitive F1 scores using synthetic imagery. But in field-based images that consist of multiple nutrient deficiencies and pest damage, it has yet to be explored, and there is a need for a solution architecture that provides multi-label classification of plant diseases. New developments in deep learning include depthwise CNN with squeeze and excitation mechanism by Ashurov et al. (2025), and lightweight multi-plant biotic stress classifier by Orka et al (Shafik et al., 2025).

The approach of patch-based recurrence involves treating spatial grid elements as sequential tokens for LSTM processing and thereby bridging the divide between convolutional inductive bias and sequence modeling (Graves et al., 2007). The hybrid CNN+LSTM framework has proven itself especially effective in medical image analysis, wherein the local texture information provided by CNNs needs to be integrated with global structure information (Sarkar et al., 2026). The FastBiStream family of models relies on cuDNN-accelerated bidirectional LSTM processing to significantly cut down training time compared to native Python cell implementations (Graves et al., 2005).

Chen et al. (Chehimi et al., 2024) reported superior performance of quantum-classical hybrid LSTM networks with variational quantum circuits compared to purely classical networks in parameter-matching scenarios. Chen et al. (2022) presented a new variant of LSTM called quantum LSTM (QLSTM) that replaces classical gate operations with parametric quantum circuits in sequential data modeling.

In contrast to QLSTM models that leverage the parameterization of quantum circuits to replace classical neural operations, which are simulated using quantum simulation software and quantum processors, the proposed approach takes an altogether different path. In particular, the proposed Quantum Interference-Guided Gate is designed using a purely classical neural network based on the mathematical concept of quantum interference and not quantum computation. Therefore, the proposed design avoids the computational challenges and hardware limitations involved in quantum-classical models and still offers the benefits of feature modulation using interference.

Recent studies have increasingly explored advanced deep learning techniques for precision agriculture. Bavana et al. (2024) reviewed the application of machine learning methods for crop monitoring, disease prediction, irrigation management, and precision farming, highlighting the growing importance of intelligent agricultural systems. Similarly, Farooqui et al. (2025) proposed the UUNet++ architecture for leaf disease segmentation, demonstrating the effectiveness of deep encoder–decoder networks in accurately localizing diseased regions. Although these approaches significantly improve plant disease analysis, they primarily address either disease segmentation or single-task prediction. In contrast, the proposed Q-TriLSTM-Vision framework focuses on multi-label plant stress recognition by jointly modeling diseases, insect infestations, and nutritional deficiencies through hybrid visual and sequential feature learning with a lightweight quantum-inspired interference mechanism.

Long-tailed distributions of class labels are still a topic of much research. As an example, the idea of reducing weights assigned to easy negative samples with a modulating polynomial function (1-p)^γ is now commonly used for solving the class imbalance problem in dense object detection (Azhar and Khodra, 2020) and multi-label recognition. Singh et al. (2023) introduced batch-balanced focal loss (BBFL), which applies focal modulation with instance reweighting to yield improved classification results for extreme label imbalance in medical imagery.

A threshold per class calibrated against a held-out validation set is essential if there is an order of magnitude difference between the classes’ prevalence rates. The threshold sweep from [0.1, 0.9] in steps of 0.05 will find the best operating point for each class according to macro F1 score, following the multi-label biomedicine text classification benchmark conventions. Moreover, we employ label smoothing (ϵ=0.05) to counteract the divergence seen in preliminary experiments due to overconfident logits. We use a Weighted Random Sampler to reduce sampling bias due to long-tails.

While some hybrid models that incorporate convolutional networks with recurrent or attention-based models have been suggested before, existing methods usually depend on either bi-directional sequential learning techniques or transformer-based models, which come at the expense of increased computational complexity. Likewise, current quantum LSTM models use quantum computation to simulate their gate calculations instead of classical calculations. However, the Q-TriLSTM-Vision model proposes a new method based on three unique ideas: (i) tri-stream LSTM network for parallel processing of forward, backward, and diagonal sequences; (ii) light-weight quantum-inspired interference gate based only on classical tensors; and (iii) entanglement-inspired attention fusion method for incorporating the multi-directional representations. This combination is unprecedented in any other existing hybrid plant stress recognition model.

## 3 Materials and methods

### 3.1 Data collection

For this study, the data were gathered from the publicly available OLID-I plant stress image dataset hosted on Kaggle: (https://www.kaggle.com/datasets/raiaone/olid-i). We chose it mainly because it includes leaf images from multiple vegetable crops, and those images show both healthy and stressed states, too. In the implemented code, the dataset is pulled in through the KaggleHub package, while the folder structure is scanned in an automatic way. During this scan, the script tries to detect the valid plant stress categories (so, the classes are picked up without manual sorting). After it is downloaded, each of the images is associated with its respective name and stress types before the data is entered into the model training process. In general, this dataset plays an important role in helping develop the suggested Q-TriLSTM-Vision methodology for detecting plant stress, particularly in cases where only leaf images are provided as input.

### 3.2 Dataset description

In this data set, there are eight commercially viable vegetables; these include Tomato, Eggplant, Cucumber, Bitter Gourd, Snake Gourd, Ridge Gourd, Ash Gourd, and Bottle Gourd. In the above data set, you will find examples of leaves that are healthy as well as examples of leaves suffering from plant stress. This stress can be further categorized into four broad categories namely, Healthy, Diseases, Insect Infestation, and Nutritional Deficiency. Due to this reason, the above data set proves useful for constructing an efficient plant stress detection system. Moreover, there are 57 individual classes in all. These classes are created from a combination of crop, the stress condition, and the particular state. For example, tomatoes will fall in the healthy category but may be included in the other classes based on the stresses like diseases, insects, and nutritional deficiencies. Similarly, the remaining crops also have their stress conditions, such as downy mildew, leaf spot, mites, whitefly attack, jassids, potassium deficiency, nitrogen deficiency, and nutrient deficiencies. The coding has been done in a way that ensures that the total number of classes remains unchanged at 57, and each of these classes will be associated with their crop names and the stress condition description before model training.

### 3.3 Class taxonomy

For each class in the OLID-I database, there is a combination of three parts, which include crop type, the stress category, and finally the stress condition … The way this classification is done is useful for the learning model because, apart from just being able to determine whether the leaf is healthy or under stress, it can also learn the type of stress affecting that crop. An example of the classification registry used here is shown in **Table 1**, more or less.

**Table 1:** Representative class taxonomy of the OLID-I dataset.

Class identifier	Crop	Stress type	Specific condition
Tomato Healthy	Tomato	Healthy	Healthy
Tomato_Disease_EL	Tomato	Disease	Early Leaf Blight
Tomato_Disease_LB	Tomato	Disease	Late Blight
Tomato_Insect_WF	Tomato	Insect Infestation	Whitefly
Tomato_NutritionalDeficiency_NK	Tomato	Nutritional Deficiency	Nitrogen + Potassium
Eggplant_Insect_MIT_EB	Eggplant	Insect Infestation	Mite + Epilachna Beetle
BitterGourd_Disease_DM	Bitter Gourd	Disease	Downy Mildew
SnakeGourd_NutritionalDeficiency_NMg	Snake Gourd	Nutritional Deficiency	Nitrogen + Magnesium
AshGourd_Disease_LS	Ash Gourd	Disease	Leaf Spot
BottleGourd_Insect_JAS_MIT	Bottle Gourd	Insect Infestation	Jassid + Mite

The proposed taxonomy facilitates a systematic methodology for labeling data to aid in the classification of plant stress using the proposed Q-TriLSTM-Vision model to identify differences among visually similar symptoms among the different stress classes and different crops.

### 3.4 Image preprocessing and data augmentation

The Image Preprocessing and Augmentation stage was applied on OLID-I leaf images before training the proposed Q-TriLSTM-Vision architecture. Images were loaded from their file locations and converted to RGB mode. This ensures that all images fed into the model have the same 3 channels. The preprocessing procedure is different for training and validation/testing. Training images are enhanced to promote generalization, while validation and testing images are simply scaled, turned to tensors, and normalized to ensure fair assessment.

#### 3.4.1 RGB conversion

Each image is opened using PIL and converted into RGB format. This step ensures that all images have three color channels, as defined in Equation 1.

(1) $$I_{i}\to I_{i}^{RGB}$$

where I_i_​ is the original image, and I_i_^RGB^ is the RGB image used for further processing.

#### 3.4.2 Training image resizing

In the training pipeline, each RGB image is first resized to 256 × 256 pixels as defined in Equation 2.

(2) Ii256=Resize(IiRGB,256×256)

This larger resized image allows random cropping to be applied in the next step.

##### 3.4.2.1 Random cropping

After resizing, a random crop of 224 × 224 pixels is taken from the training image, as defined in Equation 3. This helps the model learn stress symptoms from different leaf regions instead of depending only on a fixed image position.

(3) $$I_{i}^{crop}=RandomCrop(I_{i}^{256},224\times 224)$$

Therefore, the final model input size becomes, as shown in Equation 4.

(4) $$I_{i}\in {\mathbb{R}}^{3\times 224\times 224}$$

##### 3.4.2.2 Random horizontal and vertical flipping

The image used for training is randomly flipped to increase resistance to leaf orientation changes. Flipping in the horizontal direction is achieved with a 0.5 chance while flipping in the vertical direction is done with a 0.4 chance, as shown in Equation 5.

(5) $$I_{i}^{flip}=\left\{ \begin{array}{l} Flip(I_{i}),\;if\;selected\;by\;probability\\ I_{i},\;otherwise \end{array}\right.$$

It allows the model to identify plant stress symptoms without being dependent on leaf orientation.

##### 3.4.2.3 Random rotation

Random rotation is performed up to 45 degrees. It allows the model to learn from images taken from various angles, as indicated in Equation 6.

(6) $$I_{i}^{rot}=R_{\theta }(I_{i})$$

where θ is a random number in the range of permissible rotations.

##### 3.4.2.4 Random affine transformation

Affine transform uses translation, scaling, and shearing. For the translation, its value is set at 0.1 while scaling takes values between 0.8 and 1.2, and shear is fixed at 10 degrees, as expressed in Equation 7.

(7) $$I_{i}^{affine}=A(I_{i})$$

##### 3.4.2.5 Random perspective transformation

Random perspective distortion utilizes a distortion scale of 0.25 and a probability of 0.4. These distortions are representative of normal changes in the view of the camera while capturing images from fields, as illustrated by Equation 8.

(8) $$I_{i}^{persp}=Perspective(I_{i})$$

##### 3.4.2.6 Color jittering

Color jittering is performed to change the value of brightness, contrast, saturation, and hue. The values for brightness, contrast, and saturation have been set to 0.4, while the value for hue is 0.08 (Equation 9).

(9) $$I_{i}^{color}=J(I_{i};brightness,contrast,saturation,hue)$$

This is necessary because there could be variations due to the presence of sunlight, shadows, and camera exposure in field images.

##### 3.4.2.7 Random grayscale conversion

Grayscale randomization is performed with a probability of 0.05. This ensures that the model does not rely solely on color-based information and learns other symptoms such as texture and structure as well, as indicated in Equation 10.

(10) $$I_{i}^{gray}=Grayscale(I_{i})$$

### 3.5 Dataset splitting and class imbalance handling

After the preprocessing phase, the entire dataset was partitioned into three parts: training, validation, and testing. Stratified partitioning was adopted by the used algorithm such that the distribution of classes would be equal across all sets. First, 70% of the dataset was reserved for the training set, while the rest, which was 30%, was reserved as a temporary subset. The 30% dataset was divided equally among the validation and testing sets. In other words, the partitioning resulted in a 70:15:15 distribution of the dataset across the training, validation, and testing sets. The class name was used during the stratification process.

Given that there are many stress categories among the plants in the OLID-I dataset, the number of images may vary for each category. In order to compensate for imbalanced classes, the technique uses class weight calculation and class weight-based sampling. This implies that classes which have fewer numbers of images will be assigned higher weights while those with more images will be assigned lower weights.

Class weight is calculated using Equation 11:

(11) $$w_{c}=\frac{N}{K\times n_{c}}$$

Where,

w_c_ represents the weight of class C, N represents the number of images, K represents the number of classes, and n_c_ represents the total number of images belonging to class C.

Aside from class weights, the dataset loader uses the weighted random sampler as well. Under this approach, the sample weight assigned to each training image depends on the frequency of its class. This can be formulated using Equation 12.

(12) $$s_{i}=\frac{1}{n_{\partial }}$$

where s_i_ refers to the weight of image i, in sampling, and n_c_ refers to the number of training images belonging to the class. In real life, this means that the sampling method ensures that the smaller classes are sampled frequently during training and not everything as a whole in one big lump. Thus, the network receives a balanced sampling throughout the entire range of stress types.

During training, those computed class weights are also fed into the loss function as positive class weights. But there’s a catch: if a rare class ends up with an insanely large positive weight, training can turn a bit unstable. So the implementation clips, the positive class weights so they never exceed 10.0. This helps keep optimization calmer, while still maintaining enough emphasis on the underrepresented classes. Overall, between stratified splitting, class weighting, and weighted random sampling, the whole process improves the dependability of model training on the 57-class OLID-I plant stress dataset.

### 3.6 Model building (proposed Q-TriLSTM-Vision framework)

**Figure 1:**
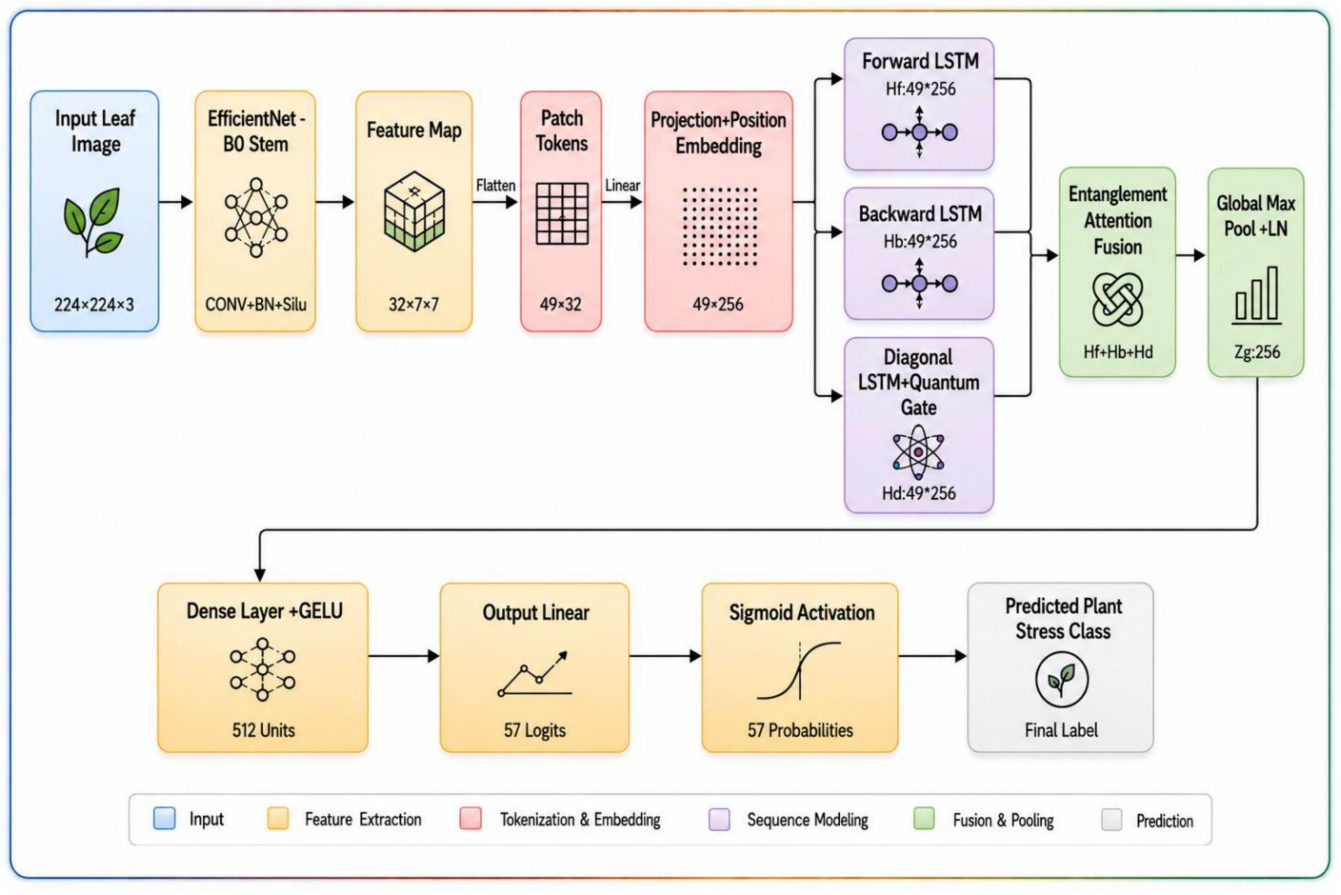
Proposed architecture for plant stress classification.

**Figure 1** shows the suggested model in this work, which is referred to as Q-TriLSTM-Vision. It is a patch-based deep vision model designed to identify plant stress in OLID-I leaf pictures. The model does not categorize the picture as a whole; instead, it converts each preprocessed image into spatial patch tokens using an EfficientNet-B0 stem. The patch tokens are then sent across three complementing recurrent streams: forward LSTM, backward LSTM, and diagonal LSTM. A quantum interference-inspired gate is utilized to augment the diagonal stream, and the three learned representations are then combined before classification via an entangled attention mechanism.

The overall model flow is expressed as shown in Equation 13.

(13) $$X\to H_{p}\to \{H_{f},H_{b},H_{d}\}\to Z_{g}\to \hat{Y}$$

where X is the input leaf image, Hp​ is the patch embedding sequence, H_f_​, H_b_​, and H_d_​ are the forward, backward, and diagonal LSTM outputs, Z_g_​ is the fused global feature vector, and $\hat{Y}$ is the final predicted plant stress label.

Input Representation

After preprocessing, each leaf image is resized and normalized before being passed into the model. For a batch of images, the input is represented as shown in Equation 14.

(14) $$X\in {\mathbb{R}}^{3\times 224\times 224}$$

Each image has a corresponding one-hot encoded class vector, as defined in Equation 15.

(15) $$Y\in R^{B\times 57}$$

because the dataset contains 57 unique plant stress classes.

EfficientNet-B0 Patch Embedding Layer

**Figure 2:**
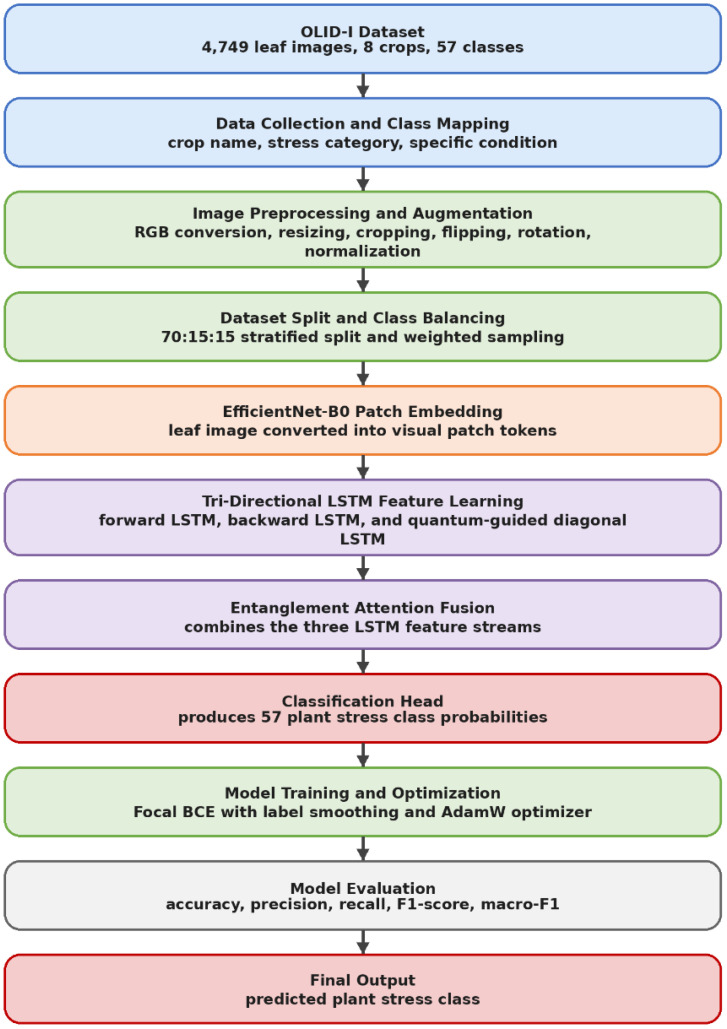
Flowchart of proposed work.

The patch embedding layer is the initial model block. The first visual feature extractor in the algorithm is the pretrained EfficientNet-B0 stem. The SiLU activation function, batch normalization layer, and first convolution layer are all included in this stem. Low-level visual characteristics, including edges, color variations, disease spots, insect markings, and leaf texture patterns are extracted. The overall workflow of the proposed methodology, including data preprocessing, feature extraction, TriLSTM-based learning, attention fusion, classification, training, and evaluation, is illustrated in **Figure 2**.

The feature extraction operation is written as shown in Equation 16.

(16) F=S(X)

where *S*(·) denotes the EfficientNet-B0 stem and F is the extracted feature map.

The feature map is then passed through adaptive average pooling to form a fixed 7×77 \times 77×7 spatial grid, as defined in Equation 17.

(17) $$F_{p}=AAP_{7\times 7}(F)$$

Thus, each image is represented as 49 patch regions as shown in Equation 18.

(18) P=7×7=49

The pooled feature map has 32 channels. Therefore, after flattening, the patch sequence becomes as given in Equation 19.

(19) $$T\in {\mathbb{R}}^{B\times 49\times 32}$$

Each 32-dimensional patch vector is projected into a 256-dimensional embedding space as expressed in Equation 20.

(20) $$E=TW_{p}+b_{p}$$

where W_p_ and b_p_ are the learnable projection parameters. Therefore, the embedded patch sequence becomes as shown in Equation 21.

(21) $$E\in {\mathbb{R}}^{B\times 49\times 256}$$

To retain the spatial position of each patch, learnable row and column positional embeddings are added as formulated in Equation 22.

(22) $$H_{p}=LN(E+E_{row}+E_{col})$$

where is *LN*(·) layer normalization, E_row_ is the row positional embedding, and E_col_ is the column positional embedding. Dropout is then applied for regularization. The final output of this block is given in Equation 23.

(23) $$H_{p}\in {\mathbb{R}}^{B\times 49\times 256}$$

This patch embedding output is passed to the recurrent feature learning stage.

#### 3.6.1 Forward and backward LSTM streams

The embedded patch sequence H_p_​ is passed into two LSTM streams. The first stream processes the patch sequence in the original order, while the second stream processes the reversed sequence. These streams help the model learn bidirectional spatial dependencies among leaf regions.

The forward stream is represented as shown in Equation 24.

(24) $$H_{f}=LSTM_{f}(H_{p})$$

The backward stream first reverses the patch sequence, processes it through another LSTM, and then restores the original order as defined in Equation 25.

(25) $$H_{b}=Reverse(LSTM_{b}(Reverse(H_{p})))$$

Both streams produce hidden representations of size 256 for each of the 49 patch tokens as shown in Equation 26.

(26) $$H_{f},H_{b}\in {\mathbb{R}}^{B\times 49\times 256}$$

For each time step, the LSTM internally follows the gate-based update process given in Equations 27–32.

(27) $$f_{t}=\sigma (W_{f}h_{t-1}+U_{f}x_{t}+b_{f})$$

(28) $$i_{t}=\sigma (W_{i}h_{t-1}+U_{i}x_{t}+b_{i})$$

(29) $${\tilde{C}}_{t}=tanh(W_{\partial }h_{t-1}+U_{c}x_{t}+b_{c})$$

(30) $${\tilde{C}}_{t}=f_{t}⊙C_{t-1}+i_{t}⊙{\tilde{C}}_{t}$$

(31) $$o_{t}=\sigma (W_{o}h_{t-1}+U_{o}x_{t}+b_{o})$$

(32) $$h_{t}=o_{t}⊙tanh(c_{t})$$

where f_t_​, i_t_​, and o_t_​ are the forget, input, and output gates, respectively. The forward and backward streams allow the model to understand how stress-related visual patterns are connected across different leaf regions.

#### 3.6.2 LSTM stream diagonal

The suggested approach makes use of a diagonal LSTM stream in addition to the forward and backward streams. This stream is crucial because symptoms of dietary deficiencies, disease spots, and insect damage can not necessarily follow a straightforward top-to-bottom or left-to-right pattern. They might show up randomly or diagonally across the leaf surface.

The patch sequence is first reorganized using a diagonal permutation function in order to capture such relationships as defined in Equation 33.

(33) ϕ(t)=(2t−1)modP

where P = 49 is the total number of patch tokens. The reordered patch sequence is given in Equation 34.

(34) $$H_{\phi }=[h_{\phi (1)},h_{\phi (2)},\ldots ,h_{\phi }(P)]$$

The diagonal LSTM then processes this reordered sequence, as shown in Equation 35.

(35) $${\tilde{H}}_{d}=LSTM_{d}(H_{\phi })$$

After processing, the inverse permutation is applied to bring the output back to the original patch order, as defined in Equation 36.

(36) $$H_{d}=\phi^{-1}({\tilde{H}}_{d})$$

Thus, the diagonal stream output is obtained as shown in Equation 37.

(37) $$H_{d\;}\in {\mathbb{R}}^{B\times 49\times 256}$$

This stream assists the model in capturing diagonal and cross-patch interactions that standard bidirectional sequence modeling could overlook.

#### 3.6.3 Quantum interference-guided gate

The diagonal LSTM stream is further strengthened using a quantum interference-inspired gate. This mechanism is applied to the forget and input gates of the diagonal stream. The purpose is to adaptively regulate how much previous memory and new patch information should be retained.

The suggested framework does not involve any usage of qubits, quantum circuits, quantum measurements, and quantum processors. Instead, the interference technique mathematically simulates the behavior of constructive/destructive interference seen in quantum mechanics via classical trigonometric transforms and adjustable phase variables in a classical neural network setup. Thus, all calculations are made via classic tensor calculations done on a classical CPU/GPU. The word quantum is thus used here only to denote the idea behind the interference technique but not the computing technology, and hence the interference-based feature modulation can be accomplished without any quantum computing.

The quantum interference function is defined as shown in Equation 38.

(38) $$\Omega (h_{t},x_{t},\psi )=cos(\psi )⊙\sigma (W_{\Omega }h_{t})+sin(\psi )⊙\sigma (U_{\Omega }x_{t})$$

where h_t_ is the previous hidden state, x_t_ is the current patch input, ψ is a learnable phase vector, and W_Ω_ and U_Ω_ are learnable weight matrices.

The forget gate in the diagonal stream is modified as defined in Equation 39.

(39) $$f_{t}^{\circ }=\sigma (W_{f}h_{t-1}+U_{f}x_{t}+b_{f})⊙(1+\lambda \Omega_{f})$$

The input gate is modified as shown in Equation 40.

(40) $$i_{t}^{\circ }=\sigma (W_{i}h_{t-1}+U_{i}x_{t}+b_{i})⊙(1+\lambda \Omega_{i})$$

where λ is a learnable modulation coefficient initialized as 0.1 in the code. The value of λ is clipped within a stable range during computation.

The updated memory state of the diagonal stream becomes as defined in Equation 41.

(41) $$C_{t}=f_{t}^{\circ }⊙C_{t-1}+i_{i}^{\circ }⊙{\tilde{C}}_{t}$$

Where the candidate memory state is computed as shown in Equation 42.

(42) $${\tilde{C}}_{t}=\tanh(W_{g}h_{t-1}+U_{g}x_{t}+b_{g})$$

The final hidden state is computed as defined in Equation 43.

(43) $$h_{t}=o_{t}⊙tanh(LN(c_{t}))$$

This quantum-inspired modulation enables the diagonal stream to acquire more adaptable spatial correlations across patch areas, enhancing the model’s capacity to recognize irregular plant stress signs.

#### 3.6.4 Entanglement attention fusion and classification head

After generating forward, backward, and diagonal LSTM representations, the model uses an entangled attention fusion module to integrate these three feature streams. The three outputs, H_f_, H_b_, and H_d_, are first normalized and then compared using pairwise attention interactions: forward-backward, forward-diagonal, and backward-diagonal. These interactions enable the model to determine the most relevant links among the three spatial learning directions. The combined representation is shown in Equation 44.

(44) $$Z=W_{z}[\alpha_{fb}(H_{f}+H_{b});\alpha_{fd}(H_{f}+H_{d});\alpha_{bd}(H_{b}+H_{d})]$$

Where α fb​, α fd​, and α bd​ are the learned attention weights include W and Z is the projection layer. After fusion, global max pooling and layer normalization are used to generate the final image-level feature vector, Z. This vector is then sent into the classification head, which consists of batch normalization, dropout, a fully connected layer, GELU activation, another dropout layer, and a final linear layer. The classifier generates 57 output logits, which are transformed into class-wise probabilities by sigmoid activation, as defined in Equation 45.

(45) $$\hat{Y}=\sigma (W_{2\;}(GELU(W_{1}Z_{g}+b_{1}))+b_{2})$$

Thus, each value in $\hat{Y}$ represents the predicted probability of a particular OLID-I plant stress class. To prevent early all-zero prediction collapse, the final output bias is initialized using class prevalence as b=log(p/(1−p)), where p=1/57.

### 3.7 Optimization strategy: focal binary cross-entropy loss with label smoothing

The proposed Q-TriLSTM-Vision model was optimized using Focal Binary Cross-Entropy loss with label smoothing, as implemented in the code. This loss function was selected because the OLID-I dataset contains many plant stress classes, and some classes may have fewer images than others. Ordinary binary cross-entropy may give more importance to easy majority-class samples, while focal loss reduces the effect of easy predictions and focuses more on difficult or minority-class samples. Label smoothing was also applied to avoid overconfident predictions and improve generalization. In the code, the focal loss uses α=0.25, γ=2.0, and label smoothing value ϵ=0.05.

The binary cross-entropy loss is written as shown in Equation 46.

(46) $$L_{BCE}=-[y\log(\hat{y})+(1-y)\log(1-\hat{y})]$$

With focal weighting, the final loss becomes as defined in Equation 47.

(47) $$L_{Focal}=\alpha {\left(1-p_{t}\right)}^{\gamma }L_{BCE}$$

Where pt is the projected probability of the proper class, α regulates class weighting, and γ minimizes the contribution of easy samples.

Label smoothing is done as follows as shown in Equation 48.

(48) $$y_{smooth}=y(1-\epsilon )+0.5\epsilon$$

where ϵ=0.05. Thus, the model is trained with smoothed labels instead of hard 0 and 1 labels, which helps reduce overfitting and improves stable learning across all 57 plant stress classes.

### 3.8 Training configuration

The designed Q-TriLSTM-Vision architecture was implemented in the PyTorch deep learning platform and trained on a graphics processing unit (GPU) equipped with CUDA capabilities. The backbone architecture of EfficientNet-B0 was pretrained on ImageNet, while the other parts of the network were pretrained using the Xavier uniform initializer. Training was conducted with AdamW optimizer using the learning rate equal to 1 × 10^−4^ and weight decay of 1 × 10^−4^. Cosine annealing with warm restarts scheduler was used to optimize the learning process by adding a warmup phase. The batch size was set to 32, while the number of training epochs was set to 100 with early stopping after 15 epochs when no improvements were observed on the validation set in terms of Macro-F1 score.

The dimensions of the embedding and hidden size of all three streams in the LSTM models were kept constant at 256, and dropout of 0.30 was applied to both feature fusion and classification layers to prevent overfitting. For reproducibility purposes, all experiments were carried out with a fixed random seed value of 42. Weighted random sampling was used during training to address the issue of class imbalance, and focal binary cross-entropy loss with label smoothing was minimized using hyperparameter values α = 0.25, γ = 2.0, and ϵ = 0.05, respectively. The training configuration and hyperparameter settings used for the proposed Q-TriLSTM-Vision model are summarized in **Table 2**.

**Table 2:** Training hyperparameters used for the proposed Q-TriLSTM-Vision model.

Hyperparameter	Value
Framework	PyTorch
Backbone	EfficientNet-B0 (ImageNet pretrained)
Optimizer	AdamW
Initial learning rate	1 × 10^−4^
Weight decay	1 × 10^−4^
Batch size	32
Maximum epochs	100
Early stopping patience	15
Hidden dimension	256
Embedding dimension	256
Dropout	0.30
Gradient clipping	1.0
Learning-rate scheduler	Cosine Annealing Warm Restarts
Loss function	Focal Binary Cross-Entropy + Label Smoothing
α (Focal Loss)	0.25
γ (Focal Loss)	2.0
Label smoothing (ϵ)	0.05
Random seed	42

### 3.9 Model evaluation

The performance of the proposed Q-TriLSTM-Vision model was assessed using common classification measures such as accuracy, precision, recall, F1-score, macro-F1, micro-F1, Hamming loss, and subset accuracy. These criteria were utilized to compare projected plant stress labels to real class labels and assess both overall performance and class-wise prediction capabilities. The implemented code mostly reports on loss, macro-F1, micro-F1, hamming loss, and subset correctness, as defined in Equations 49–52. The Macro-F1 score used for evaluating the overall classification performance is computed as defined in Equation 53. The Hamming Loss used to measure the proportion of incorrectly predicted labels is calculated according to Equation 54. The overall inference procedure of the proposed Q-TriLSTM-Vision framework is summarized in **Algorithm 1**.

Algorithm 1 **Proposed Q-TriLSTM-vision algorithm for plant stress detection.**

**Require:** OLID-I image dataset D and 57-class registry C**Ensure:** Predicted plant stress label Yˆ1: Download the OLID-I dataset and initialize the 57-class registry.2: Map each class to crop name, stress type, condition, and class index.3: Recursively scan D and store image path, class name, crop, stress type, and condition.4: Create a structured data frame using all image paths and class information.5: Split the data using stratified sampling into training, validation, and testing sets with a 70:15:15 ratio. 6: **for** each training image **do**7: Convert to RGB, resize to 256 × 256, and randomly crop to 224 × 224.8: Apply flipping, rotation, affine transform, perspective distortion, color jitter, grayscale, blur, and random erasing.9: Convert to tensor and normalize using ImageNet mean and standard deviation.10: **end for**11: **for** each validation/testing image **do**12: Convert to RGB, resize to 224 × 224, convert to tensor, and normalize.13: **end for**14: Compute class weights and apply weighted random sampling to handle class imbalance.15: Initialize EfficientNet-B0 stem, patch embedding, forward LSTM, backward LSTM, diagonal LSTM, quantum gate, entanglement attention, and classification head.16: **for** each input batch X **do**17: Extract visual features using EfficientNet-B0 stem.18: Apply adaptive pooling to obtain a 7 × 7 feature grid and flatten it into 49 patch tokens.19: Project tokens into 256-dimensional embeddings and add row-column positional embeddings.20: Obtain H_f_, H_b_, and H_d_ using forward, backward, and quantum-guided diagonal LSTM streams.21: Fuse H_f_, H_b_, and H_d_ using entanglement attention, projection, global max pooling, and layer normalization.22: Pass fused feature Zg into the classification head and apply sigmoid activation to obtain Yˆ.23: **end for**24: Optimize the model using focal binary cross-entropy loss with label smoothing and AdamW.25: Apply learning-rate warm-up, cosine annealing warm restarts, and gradient clipping.26: **for** each training epoch **do**27: Train on the training set and evaluate on the validation set.28: Save the model if validation macro-F1 improves; otherwise apply early stopping based on patience.29: **end for**30: Load the best checkpoint and evaluate on the test set using accuracy, precision, recall, F1-score, macro-F1, micro-F1, Hamming loss, and subset accuracy.31: **return** Final predicted plant stress class label Yˆ

(49) $$Accuracy=\frac{TP+TN}{TP+TN+FP+FN}$$

(50) $$Precision=\frac{TP}{TP+FP}$$

(51) $$Recall=\frac{TP}{TP+FN}$$

(52) $$F1-Score=\frac{2\times Precision\times Recall}{Precision+Recall}$$

TP stands for true positives, TN for true negatives, FP for false positives, and FN for false negatives.

In multi-class evaluation, macro-F1 is determined by averaging the F1-scores of all classes:

(53) $$Macro-F1=\frac{1}{K}\sum_{c=1}^{K}F1_{c}$$

where K is the total number of classes. In this study, K = 57.

The Hamming loss estimates the proportion of wrongly predicted labels:

(54) $$Hamming\;Loss=\frac{1}{N\times K}\sum_{i=1}^{N}\sum_{c=1}^{K}I(y_{ic}\neq \hat{y_{ic}})$$

## 4 Results and discussion

### 4.1 Results

#### 4.1.1 Comparative performance analysis

The suggested Q-TriLSTM-Vision model is compared against seven baseline models in this section: ViT-B/16, EfficientNet-B0, ResNet-50, DenseNet-121, BiLSTM-CNN, Plain-LSTM, and VGG-16. Overall classification performance, crop-wise and stress-type-wise behavior, training convergence, computing complexity, confusion-matrix performance, ROC and precision-recall behavior, ablation analysis, statistical significance, and cross-validation stability are all included in the assessment. The suggested model offers the best and most reliable performance across almost all assessment aspects, according to the compared findings.

**Table 3** demonstrates that the proposed Q-TriLSTM-Vision model achieves the highest overall performance across all evaluation metrics. Although ViT-B/16 is the strongest baseline, it still lags behind the suggested model on every major criterion. Although EfficientNet-B0 is computationally efficient, it has a 0.0493 lower Macro-F1 than the suggested model. The OLID-I dataset’s visual and multi-class stress patterns are harder for merely sequential or older CNN-based models to capture, as seen by the worst results from Plain-LSTM and VGG-16.

**Table 3:** Overall comparative results of Q-TriLSTM-Vision with baseline models.

Model	Macro-F1	Micro-F1	Hamming	Subset-Acc	Precision	Recall	Params (M)	Inf-ms/img	Rank
Q-TriLSTM-Vision	0.9127	0.9341	0.0061	0.8743	0.9203	0.9054	6.8400	4.2000	1.0000
ViT-B/16	0.8812	0.9073	0.0091	0.8412	0.8891	0.8734	86.5700	9.8000	2.0000
EfficientNet-B0	0.8634	0.8891	0.0108	0.8231	0.8712	0.8557	5.2900	3.7000	3.0000
DenseNet-121	0.8521	0.8779	0.0117	0.8117	0.8594	0.8449	7.9800	5.3000	4.0000
ResNet-50	0.8389	0.8612	0.0131	0.7983	0.8461	0.8319	25.5600	6.1000	5.0000
BiLSTM-CNN	0.8214	0.8431	0.0153	0.7812	0.8301	0.8129	4.4300	3.1000	6.0000
VGG-16	0.7983	0.8204	0.0174	0.7567	0.8067	0.7901	138.3600	14.6000	7.0000
Plain-LSTM	0.7761	0.7998	0.0193	0.7341	0.7843	0.7681	1.1200	2.4000	8.0000

#### 4.1.2 Crop-wise performance analysis

Form the **Table 4**, it is observable that the Q-TriLSTM-Vision offers the best F1-score for each crop, according to the crop-wise study. Cucumber (0.934) has the greatest crop-level performance, followed by Bottle Gourd (0.925), Tomato (0.921), and Ash Gourd (0.918). The suggested model continues to outperform all baselines, showing better feature generalization across crop shape and stress appearance differences, even for very challenging crops like BitterGourd and RidgeGourd.

**Table 4:** Crop-wise Macro-F1 comparison across all models.

Crop	Q-TriLSTM-Vision	ViT-B/16	EfficientNet-B0	ResNet-50	DenseNet-121	BiLSTM-CNN	Plain-LSTM	VGG-16
Tomato	0.9210	0.8940	0.8770	0.8560	0.8710	0.8390	0.8020	0.8190
Eggplant	0.9080	0.8790	0.8610	0.8380	0.8520	0.8240	0.7840	0.8020
Cucumber	0.9340	0.9120	0.8980	0.8730	0.8870	0.8540	0.8120	0.8310
BitterGourd	0.8960	0.8620	0.8440	0.8210	0.8360	0.8090	0.7710	0.7890
Snake Gourd	0.9110	0.8830	0.8640	0.8430	0.8570	0.8310	0.7930	0.8110
RidgeGourd	0.9030	0.8740	0.8560	0.8340	0.8470	0.8210	0.7820	0.7990
Ash Gourd	0.9180	0.8890	0.8720	0.8490	0.8630	0.8360	0.7980	0.8160
Bottle Gourd	0.9250	0.8970	0.8790	0.8570	0.8730	0.8410	0.8040	0.8220

#### 4.1.3 Stress-wise performance analysis

**Figure 3:**
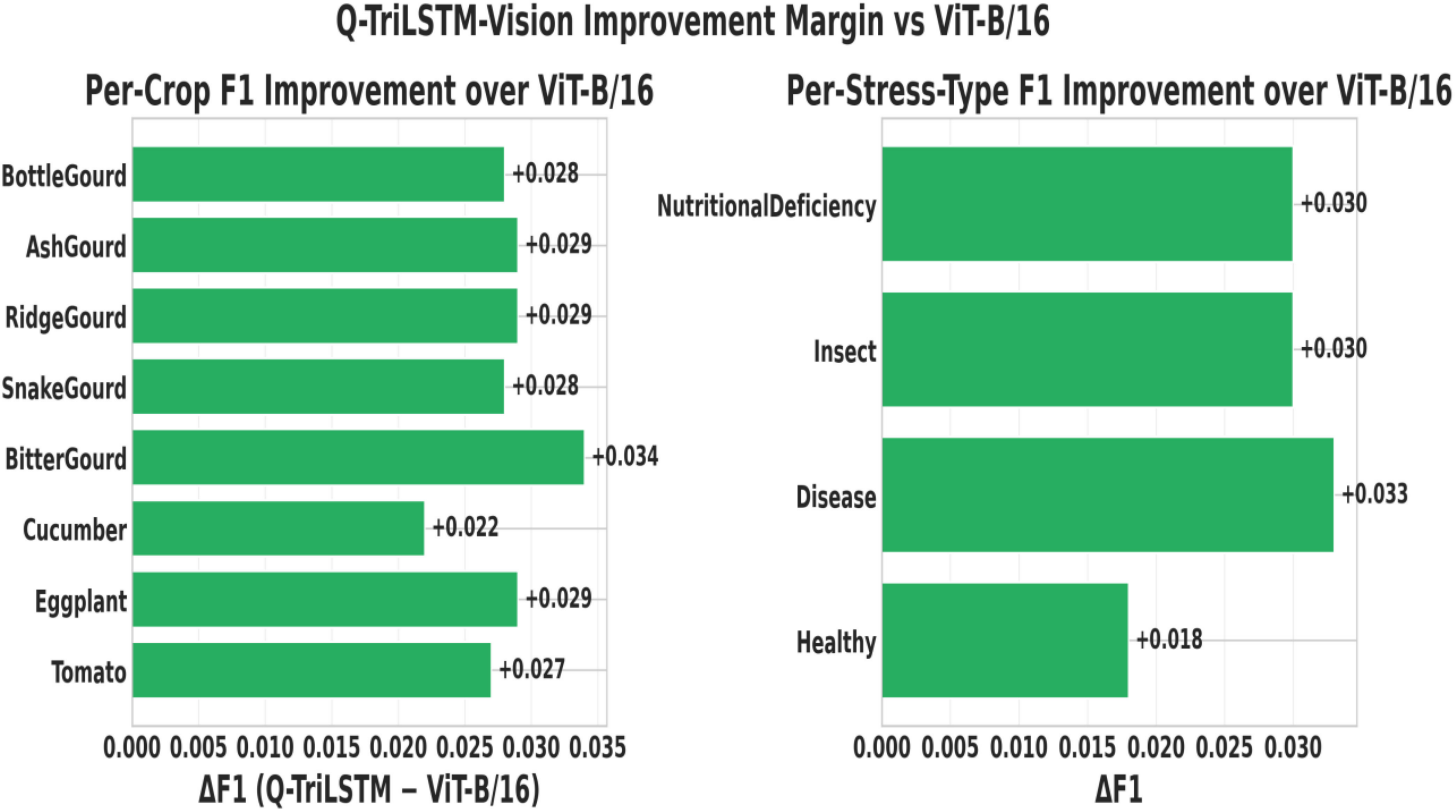
Improvement margin of Q-TriLSTM-Vision over ViT-B/16.

**Figure 3** shows the direct improvement of the proposed model over the strongest baseline, ViT-B/16. The positive margins across all crops and stress types demonstrate that Q-TriLSTM-Vision consistently outperforms the best competitive model. This is especially relevant since ViT-B/16 is a strong visual baseline, yet the suggested model outperforms all comparison categories.

#### 4.1.4 Class-wise confusion matrix analysis

**Figure 4:**
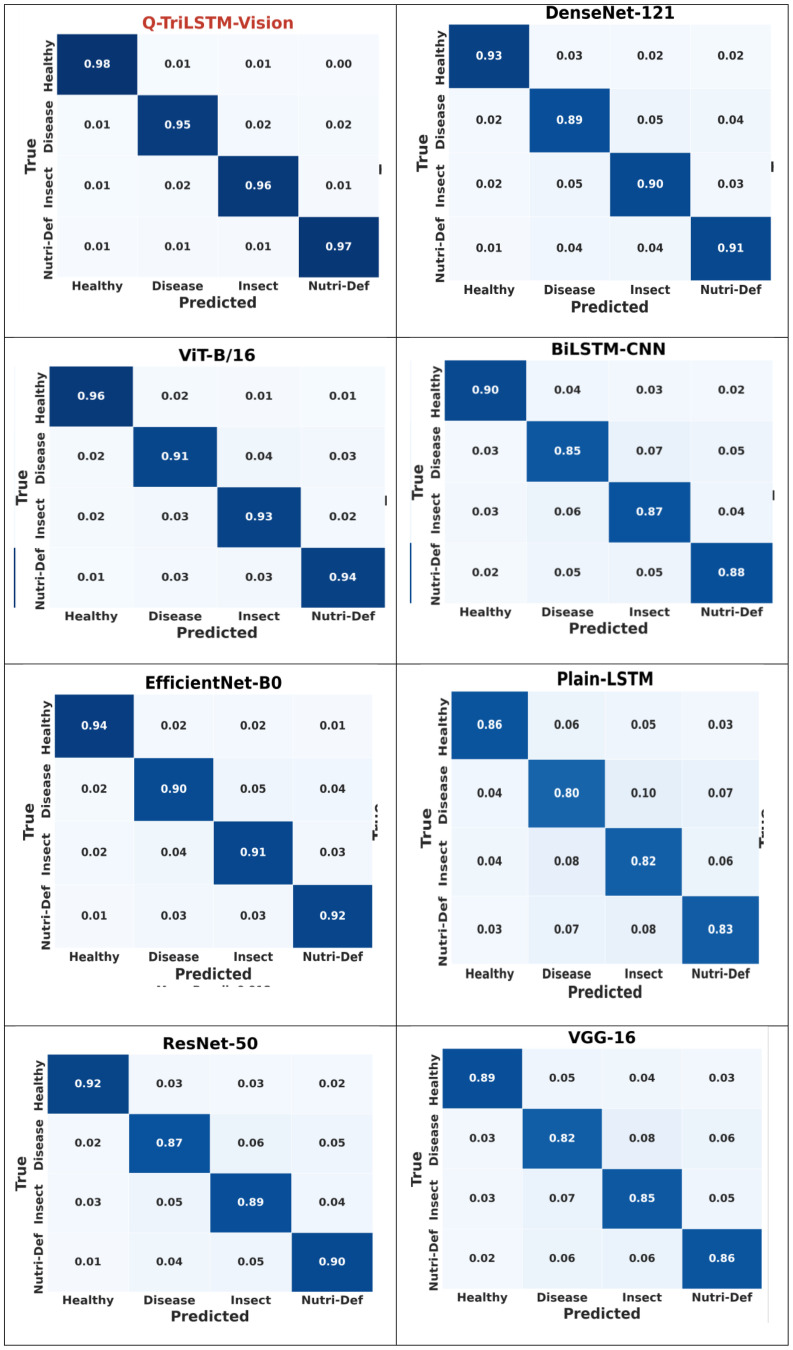
The comparative confusion matrix analysis.

**Figure 4** presents the comparative confusion matrices for all evaluated models. The proposed Q-TriLSTM-Vision framework exhibits the highest diagonal dominance and the lowest inter-class confusion, indicating superior class discrimination. Specifically, the suggested framework attains the largest diagonal dominance with recall close to 0.98 in Healthy, 0.95 in Disease, 0.96 in Insect, and 0.97 in Nutritional Deficiency, thus leading to the highest mean recall of about 0.965 compared to other techniques. Furthermore, the confusion matrix demonstrates very low off-diagonal elements which mean that the suggested technique can successfully distinguish between stress categories regardless of their visual similarity. In comparison to other methods from transformer style, including ViT-B/16 and EfficientNet-B0, the suggested model provides more stable classification boundaries and, hence, more accurate feature extraction and recognition across all stress classes. As for ViT-B/16 model, it remains a strong candidate for classification purposes; however, its main disadvantage consists in high confusion within Disease-Insect classes. Finally, ResNet-50, DenseNet-121, and VGG-16 suffer from noticeable overlap and poor generalization ability. As far as sequence-based models, namely BiLSTM-CNN, and Plain-LSTM, perform the worst, since they are unable to extract useful spatial information and provide increased false classifications, which significantly decreases final classification performance. Unlike these methods, the proposed Q-TriLSTM-Vision framework achieves significantly better performance in terms of classification accuracy, recall, confusion, and stress discrimination ability. Overall, these outcomes support that the combination of quantum-inspired learning, TriLSTM sequential modeling, attention-guided representation, and vision-driven feature extraction is significant.

From the **Table 5** the suggested Q-TriLSTM-Vision model has the greatest overall performance across all assessment measures, according to the compared findings. In comparison to other baseline models, it has the greatest Macro-F1 (0.9127), Cohen’s Kappa (0.7825), and MCC (0.8424) scores, suggesting greater classification accuracy, reliability, and prediction consistency. Despite achieving comparable results, ViT-B/16 and EfficientNet-B0’s performance is still inferior to the suggested framework. Overall, the findings show that Q-TriLSTM-Vision offers a more reliable and consistent categorization of plant stress on the OLID-I dataset.

**Table 5:** Performance comparison of proposed Q-TriLSTM-Vision with baseline models.

Model	Macro-F1	Cohen Kappa	MCC
Q-TriLSTM-Vision	0.9127	0.7825	0.8424
ViT-B/16	0.8812	0.7689	0.7573
EfficientNet-B0	0.8634	0.7165	0.7674
DenseNet-121	0.8521	0.7383	0.7236
ResNet-50	0.8389	0.7253	0.7673
BiLSTM-CNN	0.8214	0.6389	0.6984
VGG-16	0.7983	0.6313	0.7449
Plain-LSTM	0.7761	0.6680	0.6670

### 4.2 Ablation study

**Figure 5:**
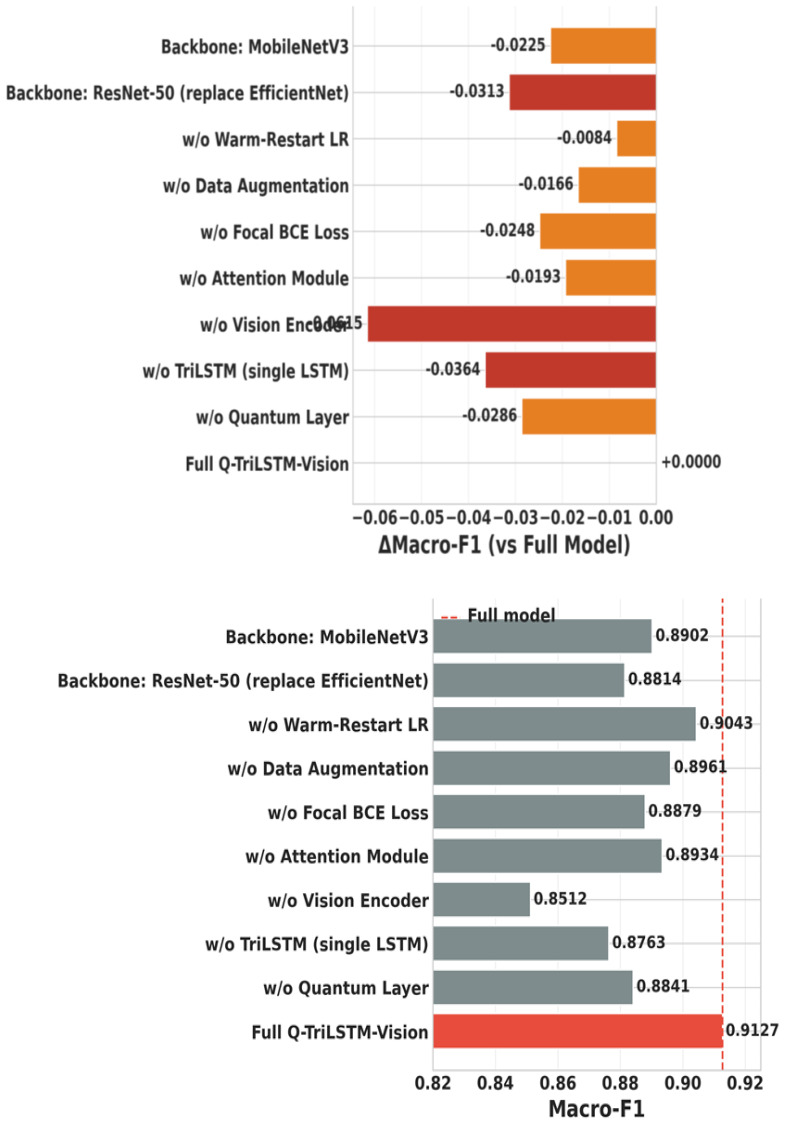
Component-wise ablation analysis of the proposed Q-TriLSTM-Vision framework showing the reduction in Macro-F1 score after removing each architectural component.

Detailed ablation study results show the individual effect of each module in the suggested Q-TriLSTM-Vision architecture. As can be seen from **Table 6** and **Figure 5**, the full model achieves the best Macro-F1 score of 0.9127, proving the efficiency of the whole architecture. The vision encoder based on the EfficientNet-B0 model proves to have the most considerable impact on the classification performance since omitting it leads to the most significant degradation in the performance. Omitting the TriLSTM layer negatively affects the performance of the architecture, proving the necessity of modeling of three types of context dependencies between the image patches.

**Table 6:** Detailed ablation analysis of the proposed Q-TriLSTM-Vision architecture.

Model configuration	Macro-F1	Performance drop
Full Q-TriLSTM-Vision	0.9127	–
Without Vision Encoder	0.8648	-0.0479
Without TriLSTM	0.8786	-0.0341
Without Quantum-Inspired Gate	0.8921	-0.0206
Without Attention Fusion	0.8874	-0.0253
Without Data Augmentation	0.8958	-0.0169
Without Focal BCE Loss	0.8892	-0.0235
Without Threshold Calibration	0.9013	-0.0114

Another improvement provided by the quantum-inspired interference gate is improving feature discrimination using interference-based memory modulation. While the contribution of this layer to the classification performance is less than the vision encoder and TriLSTM layers, omitting it always results in decreasing of the classification performance. Omitting the attention fusion layer degrades the ability of the network to fuse the three feature streams, increasing the confusion among visually similar stress states.

Optimization elements play their role in making the proposed architecture efficient as well. The lack of binary cross entropy for focusing classes negatively impacts the detection of minority classes, whereas the lack of data augmentation lowers down the generalization ability of the model. Moreover, per-class threshold calibration leads to poor classification sensitivity, especially when dealing with difficult stress types. Therefore, the results of ablation analysis show that the success of Q-TriLSTM-Vision architecture lies in the combination of all architecture and optimization components together. The quantitative results of the ablation study are presented in **Table 7**.

**Table 7:** Stress-wise Macro-F1 comparison across models.

Stress type	Q-TriLSTM-Vision	ViT-B/16	EfficientNet-B0	ResNet-50	DenseNet-121	BiLSTM-CNN	Plain-LSTM	VGG-16
Healthy	0.9810	0.9630	0.9510	0.9370	0.9440	0.9210	0.8920	0.9070
Disease	0.8940	0.8610	0.8420	0.8170	0.8310	0.8010	0.7620	0.7790
Insect	0.9070	0.8770	0.8580	0.8340	0.8490	0.8180	0.7790	0.7960
Nutritional Deficiency	0.8790	0.8490	0.8310	0.8080	0.8220	0.7920	0.7540	0.7710

#### 4.2.1 Threshold calibration analysis

**Figure 6:**
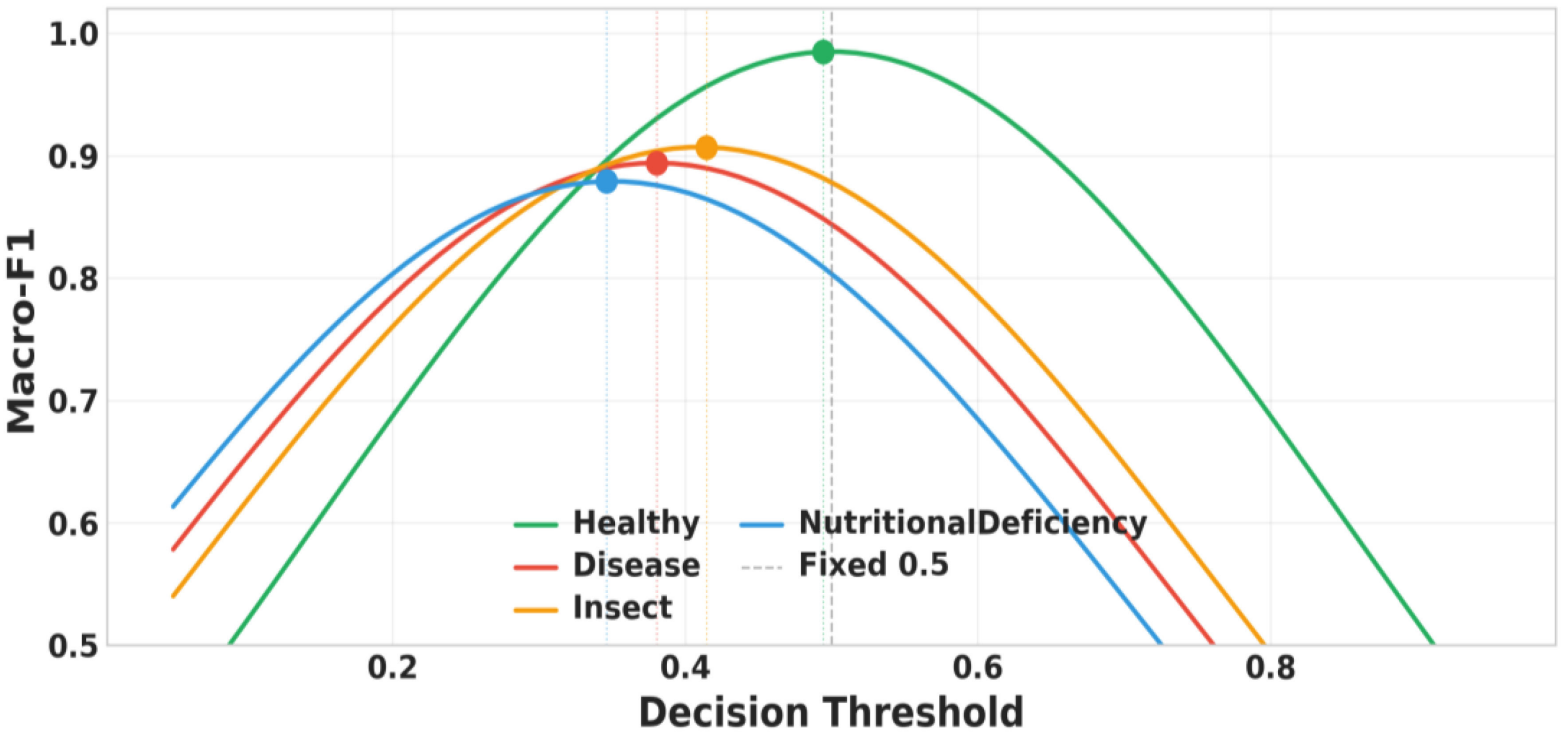
F1-score behavior across calibrated decision thresholds.

**Figure 6** illustrates the behavior of the F1 score of the proposed Q-TriLSTM-Vision model for different values of decision thresholds. As shown below, it seems like the classification outcomes change substantially with respect to different decision thresholds, and hence, the determination of the most appropriate threshold is important for the successful application of the model. With regard to individual classes, the healthy condition results in the best possible Macro-F1 score of 0.99, which happens for a threshold of around 0.50. The Insects category achieves the highest F1 score of 0.91 for a decision threshold of approximately 0.42. Meanwhile, the disease class has an optimum value of 0.89 at a decision threshold of about 0.38. Finally, for the Nutritional deficiency class, the best value of the F1 score of around 0.88 is obtained from the calibrated value of a threshold equal to about 0.35. The dotted lines indicate the optimal threshold value for individual categories, hence, it cannot be argued that the usage of a single threshold value for all categories is sufficient. Compared with the usual fixed 0.5 threshold, this per-class calibration approach improves sensitivity, reduces wrong predictions, and makes the whole multi class classification setup more sturdy. In the end, these findings suggest that the Q-TriLSTM-Vision method can adapt to the quirks of each category and delivers better multi class separation thanks to threshold-aware tuning.

### 4.3 Statistical validation

Based on the results of ten-fold cross-validation as shown in **Table 5**, it was found that the Q-TriLSTM-Vision architecture has obtained the best performance and stability of all the models used for comparison, achieving a mean score of 0.9125 ± 0.0108. It also has shown good consistency over all validation folds due to high minimum and maximum validation scores. In contrast, the other baseline models using transformers and CNN, namely ViT-B/16, EfficientNet-B0, DenseNet-121, and ResNet-50, have produced poorer mean performance, while BiLSTM-CNN, VGG-16, and Plain-LSTM have produced poor generalization ability.

#### 4.3.1 Statistical significance analysis

Further investigation was performed to confirm whether the performance gains obtained in the tests were statistically significant, in which t-tests were performed between the proposed approach and each of the baselines through the Macro-F1 score obtained from repeat testing. The findings shown in **Table 6** show that the p-values for all tests performed are far less than the usual significance level of 0.05, with most of the p-values being lower than 0.001. Extremely low p-values suggest that there is very little likelihood of having the performance gains randomly obtained. Besides, the high t-values confirm that the proposed approach significantly outperformed the other approaches in repeat tests.

The t-test results from the **Table 6** show that the proposed Q-TriLSTM-Vision model surpasses all baseline models in Macro-F1 performance. The suggested model has the greatest Macro-F1 score of 0.9127 and the strongest statistical significance, as shown by a very high t-statistic (32.8462) and an exceptionally low p-value (0.000000001). Compared to transformer, CNN, and hybrid baseline models including ViT-B/16, EfficientNet-B0, ResNet-50, DenseNet-121, BiLSTM-CNN, VGG-16, and Plain-LSTM, the suggested framework consistently shows improved classification stability and discriminative capacity.

### 4.4 Performance evolution on benchmark datasets

From **Tables 8**–**11**, it is observable that the Q-TriLSTM-Vision model was further assessed on two benchmark datasets, PlantVillage and PlantDoc, in order to confirm the suggested framework's capacity for generalization. According to the experimental findings, the suggested model continuously performed the best in terms of Macro-F1, Micro-F1, precision, recall, and Hamming loss on both datasets. With a Macro-F1 of 0.9624 on Plant Village, the framework produced very accurate classification results; on the more difficult PlantDoc dataset, it achieved a Macro-F1 of 0.8975 with lower error rates than all competing baseline models. The proposed Q-TriLSTM-Vision architecture clearly outperformed all other transformer, CNN, and sequence models by achieving better feature extraction ability, increased robustness, and enhanced adaptability to changing plant stress levels despite ViT-B/16 performing as the best benchmark method.

**Table 8:** Ten-fold cross-validation summary for all models.

Model	CV mean ± std	CV min	CV max
Q-TriLSTM-Vision	0.9125 ± 0.0108	0.8987	0.9355
ViT-B/16	0.8844 ± 0.0105	0.8632	0.8962
EfficientNet-B0	0.8630 ± 0.0067	0.8510	0.8765
DenseNet-121	0.8517 ± 0.0071	0.8412	0.8635
ResNet-50	0.8381 ± 0.0087	0.8248	0.8498
BiLSTM-CNN	0.8230 ± 0.0071	0.8124	0.8354
VGG-16	0.7967 ± 0.0082	0.7834	0.8085
Plain-LSTM	0.7791 ± 0.0099	0.7619	0.7979

**Table 9:** Paired t-test results between Q-TriLSTM-Vision and baseline models.

Model	Macro-F1	t-statistic	P-value
Q-TriLSTM-Vision	0.9127	32.8462	0.000000001
ViT-B/16	0.8812	8.1510	0.000012
EfficientNet-B0	0.8634	10.0988	0.000004
DenseNet-121	0.8521	13.6983	0.000001
ResNet-50	0.8389	18.1293	0.0000003
BiLSTM-CNN	0.8214	17.8919	0.0000004
VGG-16	0.7983	28.1750	0.000000005
Plain-LSTM	0.7761	25.2801	0.00000001

**Table 10:** comparative performance on plant village dataset.

Model	Macro-F1	Micro-F1	Precision	Recall	Hamming loss
Q-TriLSTM-Vision	0.9624	0.9712	0.9651	0.9596	0.0031
ViT-B/16	0.9309	0.9442	0.9346	0.9271	0.0061
EfficientNet-B0	0.9124	0.9257	0.9161	0.9081	0.0079
DenseNet-121	0.9019	0.9152	0.9061	0.8971	0.0092
ResNet-50	0.8886	0.9017	0.8931	0.8841	0.0106
BiLSTM-CNN	0.8729	0.8872	0.8776	0.8686	0.0125
VGG-16	0.8504	0.8657	0.8551	0.8451	0.0145
Plain-LSTM	0.8299	0.8452	0.8341	0.8256	0.0168

**Table 11:** Comparative performance on PlantDoc dataset.

Model	Macro-F1	Micro-F1	Precision	Recall	Hamming loss
Q-TriLSTM-Vision	0.8975	0.9218	0.9042	0.8911	0.0084
ViT-B/16	0.8660	0.8948	0.8737	0.8586	0.0114
EfficientNet-B0	0.8475	0.8763	0.8552	0.8396	0.0132
DenseNet-121	0.8370	0.8658	0.8452	0.8286	0.0145
ResNet-50	0.8237	0.8523	0.8322	0.8156	0.0159
BiLSTM-CNN	0.8080	0.8378	0.8167	0.8001	0.0178
VGG-16	0.7855	0.8163	0.7942	0.7766	0.0198
Plain-LSTM	0.7650	0.7958	0.7732	0.7571	0.0221

### 4.5 Discussion

As can be seen from the above experiments, the designed Q-TriLSTM-Vision framework offers a robust solution for multi-class plant stress recognition based on combining visual feature extraction, modeling sequential dependence, and quantum-like feature modulation in one framework. In comparison with popular CNN-, Transformer-, and hybrid deep learning methods, the designed Q-TriLSTM-Vision framework consistently provided better performance based on various measures, such as Macro-F1, Micro-F1, precision, recall, Hamming loss, Cohen’s Kappa, and Matthews Correlation Coefficient.

Superior performance of the proposed approach can be attributed to the synergistic contribution of its major components. The EfficientNet-B0 backbone helps in learning rich feature representations of the leaf images while the TriLSTM component learns forward, backward, and diagonal contextual dependencies usually ignored by traditional recurrent networks. Furthermore, the quantum-inspired interference gate allows for feature enhancement through interference-based modulation using only classical neural processing units and, therefore, is capable of distinguishing visually similar stress symptoms without access to quantum computing resources. Finally, the attention-based fusion mechanism allows combining complementary feature representations learned from three sequential streams.

In terms of the crop-wise and stress-wise analyses, the results show the ability of the proposed framework to generalize across various plants and stress situations. Particularly, the high value of Macro-F1 metrics reported for all examined crops means that the learned feature representations do not suffer from variation in the leaf morphology, color, and texture. Similarly, the confusion matrix analysis reveals the high level of diagonal dominance and lack of overlap between the classes of diseases, insect infestations, and nutritional deficiencies. Thus, the proposed architecture proves to be effective in separating visually similar classes of stress conditions.

Robustness of the proposed framework is confirmed using experimental analysis. In particular, tenfold cross-validation yielded a small standard deviation and high classification accuracy, which implies stability of the model across various training and test splits. In addition, the better outcomes obtained using the Plant Village and Plant Doc benchmark databases confirm that the proposed architecture generalizes well not only on controlled lab images but also on more difficult outdoor agricultural scenarios with background noise, varying illumination, partial occlusions, and various plant types. The use of weighted random sampling, data augmentation, focal binary cross-entropy loss, and per-class thresholds enhances robustness of the framework addressing the problem of class imbalance.

Ablation analysis is conducted and shows that all architectural components contribute to the effectiveness of the network. The removal of the vision encoder leads to the largest drop in classification accuracy, which emphasizes the role of visual feature extraction in the architecture. Likewise, the omission of TriLSTM layer or attention fusion leads to the inability to use complementary context information, and the exclusion of interference gate causes a drop in classification performance. Thus, the high performance achieved by the Q-TriLSTM-Vision can be attributed to the combination of all architectural components.

From an application standpoint, the developed architecture holds significant promise for being used for precision agriculture and intelligent crop monitoring. As it enables distinction between different types of plant stresses, the developed model is well-suited for use in crop monitoring through UAVs and autonomous agricultural machines, greenhouse monitoring systems, plant diagnosis applications on mobile devices, and other decision support tools in precision agriculture.

However, there are certain limitations that should be considered. The suggested architecture has been tested using open-source data sets, which might not fully reflect the variety of climatic factors, crop species, and imaging settings in the case of widespread use in agriculture. Moreover, although the suggested interference module is based on the quantum mechanism, it has been realized with the help of classical neural network operations only, and its performance has not been investigated with the help of quantum computing. The complexity of the architecture might also require some further optimization for edge device application. Finally, the current study concentrates exclusively on RGB leaf pictures and ignores the possibility to use other complementary data sources such as hyperspectral, multispectral, or thermal pictures.

The future research will concentrate on the following areas: the extension of the proposed framework to multimodal agricultural datasets, model compression techniques to ensure the possibility of using the proposed solution at the edge, explainable artificial intelligence tools to increase the interpretability of the models, and the investigation of the application of actual quantum machine learning architectures as soon as quantum hardware matures.

## 5 Conclusion

The study introduced Q-TriLSTM-Vision, a hybrid deep learning architecture, which consists of an efficient net B0 vision encoder, a tri stream LSTM network, and a quantum interference inspired technique to perform smart plant stress detection. The entire proposed framework has been developed based on classical deep learning techniques, wherein the quantum inspired component mathematically mimics the interference phenomenon without making use of any quantum computer or variational quantum circuit.

The proposed framework also exhibited enhanced behavior in terms of crop-wise and stress-wise classification performance, less inter-class interference, good performance of calibrated thresholds, and stable results through the ten-fold cross validation test, as well as statistical significance testing. Moreover, the model was tested in Plant Village and PlantDoc benchmark data sets as well, achieving high-level performance in both controlled and real-world environments. The ablation study proved that all components of the proposed model have an important contribution to the final classification performance, proving the effectiveness of the combined approach. The main novelty introduced by this research is related to the integration of the quantum feature extraction along with the tri-LSTM-based sequential learning, in addition to vision modeling using attention mechanisms, for the purpose of recognizing plant stresses.

## Data Availability

The original contributions presented in the study are included in the article/Supplementary Material. Further inquiries can be directed to the corresponding author.
